# Direct Write, Read, and Erase of a Vertical Heterostructure of Graphene–Monolayer Electrolyte–h‐BN Using Electric Force Microscopy

**DOI:** 10.1002/smll.202511129

**Published:** 2026-02-20

**Authors:** Huiran Wang, Shubham Sukumar Awate, Ke Xu, Susan K. Fullerton‐Shirey

**Affiliations:** ^1^ Department of Chemical and Petroleum Engineering University of Pittsburgh Pittsburgh Pennsylvania USA; ^2^ School of Physics and Astronomy Rochester Institute of Technology Rochester New York USA; ^3^ School of Chemistry and Material Science Rochester Institute of Technology Rochester New York USA; ^4^ Microsystems Engineering Rochester Institute of Technology Rochester New York USA; ^5^ Department of Electrical and Computer Engineering University of Pittsburgh Pittsburgh Pennsylvania USA

**Keywords:** 2D materials, atomic force microscope, electric double layer, field effect transistor, iontronics, nonvolatile memory

## Abstract

Electric double layer (EDL) gating effectively modulates charge density in two dimensional (2D) crystals; however, the electrolyte is typically deposited on top of the 2D crystal, controlling charge most strongly in the 2D material it contacts directly. Here, a monolayer‐thick electrolyte is positioned between two, 2D materials – hexagonal BN (h‐BN) and multi‐layer graphene – in a vertical heterostack. Ions in the electrolyte are bistable, meaning they can be moved by field‐effect closer to either of the 2D crystals and retain doping after the voltage is removed. Electric force microscopy (EFM) is used to write, read, and erase the heterostacks, and map the graphene surface charge with 12 × 12 nm resolution. With graphene as the top layer of the heterostack, ions in the monolayer electrolyte dope the 2D crystal from underneath, inducing sheet densities of 5 × 10^12^ $cm^{-2}$, more than half of which is retained for 25 min after the voltage is removed, confirming bistability of the monolayer electrolyte. While the doping can also be erased, the data suggest that the energy barrier to erasing is higher than to writing, resulting in partial erasure at a voltage equivalent to the write voltage but opposite in polarity. This observation is consistent with prior device measurements of the monolayer electrolyte on 2D crystal field‐effect transistors (FET).

## Graphical abstract

This work demonstrates bistable graphene doping of about 5 × 10^12^ $cm^{-2}$ using a molecularly thin electrolyte confined between two, 2D crystals. The electrolyte's bistability enables multiple non‐volatile states with a conductive AFM tip writing, erasing, and sensing these states. This approach promises scalable, nanometer‐resolution ion‐gating within 2D heterostructures for future device miniaturization.

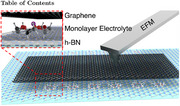

## 1 Introduction

2D crystals consist of atomic or molecular layers that are held together by van der Waals forces. Due to quantum confinement [1], these materials exhibit electrical [2, 3], magnetic [4, 5], mechanical [6, 7], and optical [8, 9] properties that are different from the bulk. Some of these novel properties have been accessed using polymer electrolytes or ionic liquids to gate 2D crystal FETs with large electric fields (∼V/nm) [3, 5, 7, 9, 10, 11]. The electrolyte is deposited on the channel of the FET, and ions are drifted by the gate to the 2D crystal interface. There, the ions induce image charges in the channel, forming an EDL that can be considered a sub‐nanometer gap capacitor with capacitance densities in the range of 1 – 10 μF $cm^{-2}$ [12, 13, 14], corresponding to sheet densities on the order of 10^13^ to 10^14^ $cm^{-2}$ [12, 15].

The molecularly thin nature and weak inter‐layer bonding of 2D crystals facilitate integration of heterostructures and van der Waals assemblies [16]. These assemblies give rise to stacks with tailored electronic and optoelectronic functionalities [17, 18]. Lateral heterostructures, in which two or more 2D crystals are combined in the plane of the channel, can be effectively gated with ions because the electrolyte is in direct contact with multiple 2D cyrstals in the channel [19, 20]. This contrasts vertical heterostructures (i.e., stacks of multiple 2D crystals), where an electrolyte deposited on top of the heterostructure contacts only the uppermost 2D crystal. Because electrolyte gating is an interfacial phenomenon, it modulates the charge most effectively in the 2D crystal it directly contacts, leaving the 2D crystals that are out of contact largely unaffected [21]. Although solid polymer electrolytes and ionic liquids can strongly modulate charge, they cannot be inserted between 2D layers in a vertical heterostructure. The ionic liquid is dropped onto the surface, forming a liquid dome over the device; and solid polymer electrolytes use high molecular‐weight polymers (>100 000 g/mol) with film thicknesses typically ranging from hundreds of nanometers to microns. Regardless of the electrolyte type or location, the gating is always volatile at room temperature – meaning that ions drift back into the electrolyte once the gate voltage is removed [22, 23].

Here, we demonstrate a vertical heterostructure in which a monolayer‐thick electrolyte is integrated between two, 2D crystals and provides non‐volatile doping at room temperature. Unlike ionic liquids or polymer electrolytes, the monolayer electrolyte is comprised of flat molecules that organize into an ordered array on 2D surfaces with a thickness of 0.46 ± 0.01 nm [24]. It comprises a cobalt phthalocyanine with four, 15‐crown‐5 ethers (CoCrPc). According to density functional theory (DFT), each crown ether solvates one cation and also presents an energy barrier to cationic movement through the crown ether cavity [25]. This energy barrier is sufficiently large at room temperature to prevent cations from moving from one side of the molecule to the other, but can be lowered by an applied electric field [25]. Thus, cations can be stable in two states – one on either side of the crown ether plane – positioning them near either one of the two, 2D crystals. If charge is supplied to the 2D crystal, an EDL forms between the cations and the induced charge, which persists even after the bias is removed [26, 27]. Thus, such a bistable electrolyte of thickness commensurate with the nearby 2D materials provides a means to dope 2D crystals from within a 2D heterostack.

In our prior work, the monolayer electrolyte was used to demonstrate non‐volatile doping of 2D crystals located underneath the electrolyte in a FET structure where ions were configured using a global backgate [26, 27, 28]. In these FETs, the monolayer electrolyte was deposited onto the 2D channel, and doping was monitored by shifts in the Dirac point of graphene [26] and the threshold voltage of WSe_2_ [27, 28]. When the monolayer electrolyte was capped with h‐BN, a WSe_2_ channel (length and width of 10 × 3 μm) showed a large ON/OFF ratio (>10^4^), high charge density (2.5 × 10^12^ $cm^{-2}$), and more than 6 h state retention (maximum time measured) [27]. However, the symmetry and bistability of the monolayer electrolyte suggests the possibility of bidirectional doping in a vertical heterostack; that is, the monolayer electrolyte should electrostatically dope a 2D crystal positioned either beneath or above it. Such a centrally placed, locally programmable dielectric could enable new device functionalities such as reconfigurable vertical junctions [29, 30], tunable interlayer tunneling or excitonic behavior [31], and vertical‐synaptic transistors [32]. While lithographic processing of vertical FETs containing a molecular monolayer requires process development, an essential first step is demonstrating that the monolayer electrolyte can also dope a 2D crystal placed on top of it.

In this work, we use EFM to write, read, and erase a vertical heterostack consisting of graphene, monolayer electrolyte and h‐BN. Graphene is chosen as the 2D material for its ambipolarity and sensitivity to nearby charge. EFM is particularly suited for this study because, unlike transport measurements, it enables mapping of surface charge with 12 × 12 nm resolution without the need for lithographic contacts. Although graphene is expected to be equipotential, meaning that doping is not expected to be localized to the region of the tip, programming and erasing with a nanosized electrode represents a step toward localized switching. This is especially relevant to the monolayer electrolyte because the molecular configurations that give rise to the non‐volatile doping are encoded in the molecules themselves. Note that charge injection and sensing with EFM is not new – it has been demonstrated in monolayers of pentacene [33], pili proteins [34], and quantum dots [35]. Here, EFM is used to demonstrate that a 2D crystal positioned on top of the monolayer electrolyte can undergo non‐volatile doping. The fact that a nanoscale contact is used provides promise for device scaling with a nanosized gate contact. Furthermore, the results confirm the non‐volatility imparted by the monolayer electrolyte, which until now has been limited to demonstration in 2D FETs.

## 2 Results and Discussion

**Figure 1:**
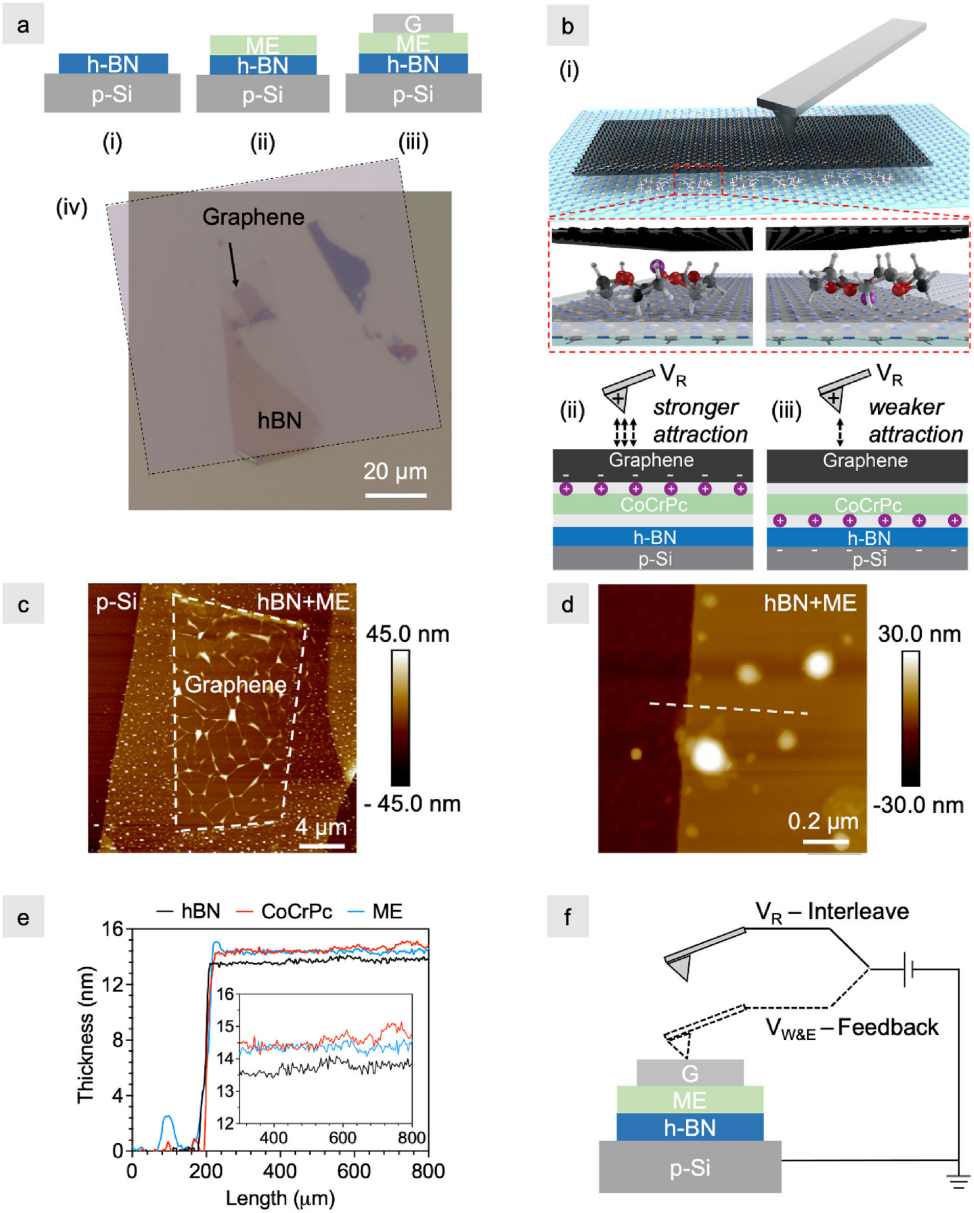
Assembly and characterization of heterostacks. (a) Stack assembly: (i) h‐BN is exfoliated onto p‐Si followed by (ii) drop‐casting and annealing the monolayer electrolyte (ME) and (iii) capping with graphene (G) using a dry flake transfer. (iv) False color optical image of the transferred graphene flake overlaid onto the monolayer‐electrolyte covered h‐BN flake. (b) Schematic of (i) the heterostack with the monolayer electrolyte in two energetically favorable configurations corresponding to the cation positioned near and far from graphene. The lower panels in (i) highlight the two crown ether/ion configurations calculated by DFT [25]. They correspond to (ii) where ${\mathrm{Li}}^{+}$ ions are near graphene, resulting in a stronger tip‐to‐sample attraction using a positive read bias, $V_{R}$, and (iii) where the ions are near h‐BN, resulting in a weaker interaction. (c) AFM scan of Stack 1 (S1). (d) Zoomed‐in AFM scan of S1 after depositing the monolayer electrolyte (ME) but before capping h‐BN with graphene; the dashed line corresponds to the location of line scans shown in (e) of the bare hN flake, after CoCrPc deposition, and after ME deposition (i.e., CoCrPc + LiClO_4_). (f) EFM schematic showing the tip in contact with graphene (dashed lines) to write ($V_{W}$) and erase ($V_{E}$) during the forward scan, and out of contact (solid lines) to read the state ($V_{R}$) during the reverse scan.

Assembly of a two‐terminal heterostack on p‐doped Si is depicted in Figure 1a(i–iii), with fabrication details provided below in the Experimental Section. Heterostack assembly and EFM characterization are performed in an argon‐filled glovebox. The monolayer electrolyte is drop‐cast onto exfoliated h‐BN, and capped with multi‐layer graphene (2 – 3 nm) using dry‐flake transfer. A representative heterostack is shown by overlaying a false‐color optical image of transferred graphene onto the electrolyte/h‐BN (Figure 1a(iv)). A cross‐sectional schematic, Figure 1b(i), depicts the ordered array of CoCrPc molecules and – in accordance with DFT calculations [25, 36] – the ion position within the crowns. The monoelectrolyte mechanism and tip‐to‐sample interactions are depicted in Figure 1b(ii, iii). While the location of the anion has not been experimentally verified, it is likely proximate to the cobalt atom of the phthalocyanine; we recently showed that salts with anions other than $\mathrm{Cl}{O_{4}}^{-}$ also show bistable switching [28]. An AFM topography scan of the stack is shown in Figure 1c, where the raised features on graphene with large lateral aspect ratio are wrinkles and polycarbonate residue from the dry flake transfer. A second, smaller AFM scan at the edge of the monolayer electrolyte‐covered h‐BN flake is shown in Figure 1d. The raised circular features are aggregates of the monolayer electrolyte with a diameter ≤100 nm, consistent with our previous observations of the monolayer electrolyte deposited on graphene [24, 26], WSe_2_ [27, 28] and MoS_2_ [27]. Linescans of the uncapped portion of the stack are used to confirm the thickness of the monolayer electrolyte as ∼1 nm (Figure 1e), consistent with previously measured values [24].

EFM is used in contact to write and erase the monolayer electrolyte, and lifted out of contact to read the state (Figure 1f). EFM is a two‐terminal, dual‐path technique that simultaneously measures topography and surface charge. Electric field gradients within the sample induce a force on the tip, giving rise to a change in the oscillation frequency of the tip with faster (slower) oscillations indicating repulsion (attraction). In the present work, bistability of the monolayer electrolyte is sensed as a change in the interaction strength between the tip and graphene that persists even after the write voltage is removed.

The following protocol is used to write, read, and erase the stack with p‐type Si (electrode 1) set to ground and voltage applied to the AFM tip (electrode 2). In the forward line scan, the tip is in contact with the graphene (tapping mode) and a non‐zero voltage is applied for writing ($V_{W}$) and erasing ($V_{E}$). On the reverse line scan, the tip is lifted to a height (Z) 20 nm above the surface of graphene, and a read bias ($V_{R}$) is applied to read the state. The read step occurs ∼1.4 s after the write step is completed, and 256 data points are collected with each line scan. For a 3 × 3 μm scan, the tip is moved 11.7 nm, and the forward and reverse scans are repeated for 256 lines. Histograms for each write and erase scan are created with 256 × 256 phase shift data points. Additional details of the EFM measurements are described in Section S1, including lift height calibration in Figure S1.

**Figure 2:**
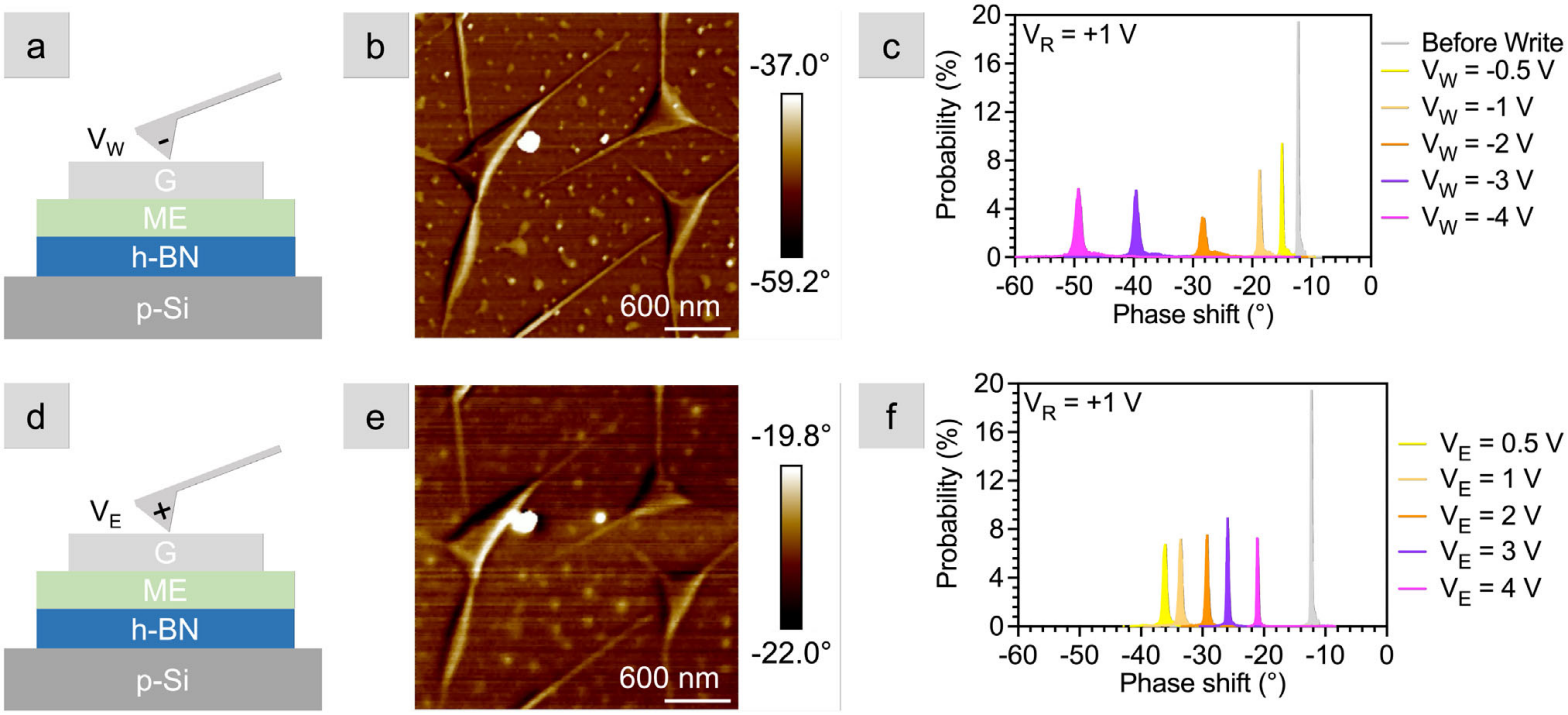
EFM measurements of the G/ME/h‐BN Stack 1, location 1 after write and erase. (a) Cross‐sectional schematic of the stack undergoing a negative write scan. (b) 3 × 3 μm phase shift image of a portion of the heterostack written with −4 V and read with +1 V. (c) Histogram of the graphene phase shift in response to a series of write voltages. (d) Cross‐sectional schematic of the stack undergoing positive erase scan. (e) 3 × 3 μm phase shift image of a portion of the heterostack erased with +4 V and read with +1 V. (f) Histogram of the graphene phase shift in response to a series of erase voltages. Z = 20 nm.

First we start with the heterostacks containing the monolayer electrolyte, with the top (bottom) rows of Figure 2 corresponding to writing (erasing). Figure 2a shows a stack schematic with a negative tip voltage, and Figure 2b is a representative scan of the phase shift at $V_{R}$ = +1 V after writing at $V_{W}$ = −4 V. The scan shows that the phase shift is negative, with a peak ∼$-50^{\circ }$, indicating a decrease in the oscillation frequency and therefore an increased attraction between the tip and the graphene surface. This value of $-50^{\circ }$ is noteworthy because the phase shift prior to writing was only ∼$-12^{\circ }$ (Figure 2c). The negative phase shift “before write” can be attributed to the difference in work functions between the PtIr tip and the n‐type graphene/CoCrPc [26] in combination with a +1 V read bias. In total, as the write voltage increases from −0.5 to −4 V, the phase shifts ∼35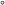 in the negative direction. The fact that the negative phase shift persists after writing indicates that the mechanism responsible for increasing the attraction between the tip and the surface is nonvolatile – at least for the time between write and read scans (∼1.4 s). Notice that the phase shift to ∼$-50^{\circ }$ is not the cumulative result of applying progressively larger voltages; rather, the same shift can be achieved by directly applying $V_{W}$ = –4 V as shown in Figure S2. Furthermore, the large write bias does not induce topographical defects on the graphene surface (Figure S3).

Next, a complementary series of positive erase voltages are applied, as shown schematically in Figure 2d. After erasing at $V_{E}$ = +4 V, a scan is taken at the same location as the write step with $V_{R}$ = +1 V (Figure 2e). On erasing, the phase shift moves toward a less negative value ∼$-20^{\circ }$, meaning that the tip is less attracted to the surface. The histograms in Figure 2f show the progressive decrease of the phase shift as the erase voltage increases from 0.5 to 4 V. In total, the phase shifts 35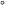 in the negative direction on writing (i.e., increasing attraction), and 30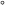 in the positive direction on erasing (i.e., decreasing attraction). Because the phase shift in response to erasing is smaller than the shift for writing, this result implies partial erasing – an observation that is discussed in more detail below.

One possible interpretation of the phase shifts is that negative write voltages attract cations toward the graphene surface, doping it more n‐type (Figure 1b(ii)). Because the read voltage is positive, this doping increases the attraction between the tip and the graphene surface, consistent with a negative phase shift. This mechanism agrees with our understanding from DFT modeling of the monolayer electrolyte [25] and transport measurements of 2D crystal monolayer electrolyte gated FETs [26, 27]. The gradual and monotonic phase shift suggests a possible distribution of energy barriers to toggle the ions through the crown ethers. That is, as more voltage is applied, additional ions move through the crown ether cavities and increase doping of the graphene surface. We observe similar behavior in FETs in which progressively larger programming voltages increase the ON/OFF ratio [27]. An analogous explanation can be applied to the erase procedure: the tip becomes less attracted to the graphene as positive erase voltages push the cations away from the graphene surface, causing it to be less n‐doped (Figure 1b(iii)).

**Figure 3:**
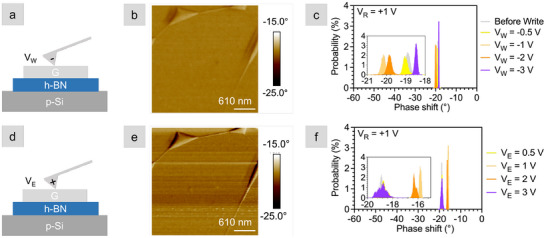
EFM measurements of G/h‐BN control Stack 1 (i.e., without the monolayer electrolyte) after write and erase. (a) Cross‐sectional schematic of the stack undergoing a negative write scan. (b) 3 × 3 μm phase shift image of a portion of the heterostack written with −3 V and read with +1 V. (c) Histogram of the graphene phase shift in response to a series of write voltage. (d) Cross‐sectional schematic of the stack undergoing a positive erase scan. (e) 3 × 3 μm phase shift image of a portion of the heterostack erased with +3 V and read with +1 V. (f) Histogram of the graphene phase shift in response to a series of erase voltages. Z = 20 nm.

To confirm that the phase shifts are caused by the monolayer electrolyte, we repeated the same write‐read and erase‐read measurement sequence, but for a graphene/h‐BN heterostack. These control measurements, shown in Figure 3, mirror the monolayer electrolyte stack in Figure 2. For both area scans (Figure 3b,e), read at $V_{R}$ = +1 V after $V_{W}$/$V_{E}$ = ±3 V, the phase shift of the control stack is nearly unchanged from the initial value of ‐19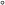 Figure 3c,f. That is, writing and erasing up to ±3 V resulted in phase shifts less than 2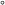 for writing and 3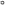 for erasing. The phase shifts are 15 and 10 times smaller than those measured for the heterostacks containing the monolayer electrolyte written at –4 and 4 V, respectively, indicating that charge injection and withdrawal are volatile in the absence of the monolayer electrolyte. The phase shift scans used to generate the histograms in Figures 2 and 3 are provided in Figures S4 and S5, along with measurements of additional heterostacks (Figures S6 and S7). The same trend is observed for all heterostacks: increasing the write voltage results in a negative phase shift, and increasing the erase voltage moves the phase shift back toward the original value.

**Figure 4:**
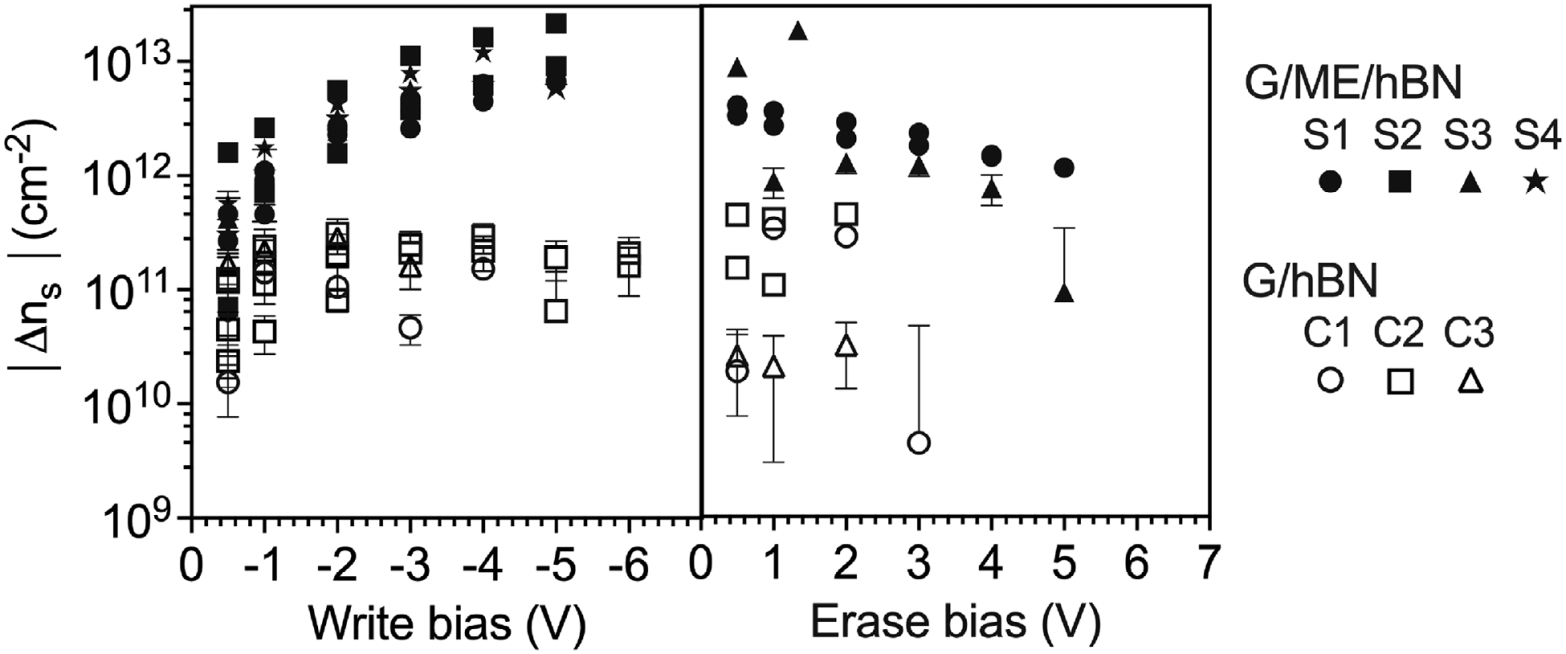
Absolute change in sheet carrier density ($\left|\Delta n_{S}\right|$) vs. bias for heterostacks without (open symbols, C1 – C3) and with (close symbols, S1 – S4) the monolayer electrolyte. The shape of the symbols distinguishes between individual heterostacks, and the repeated symbol shapes represent various locations on the same heterostack. The error bars represent one standard deviation from the mean peak value. Note that the data from Figure 2 correspond to Stack 1 (S1) Location 1, and Figure 3 data correspond to Stack 1 (C1)

We previously reported sheet carrier densities in monolayer electrolyte‐gated graphene FETs of 3.0 ± 0.2 × 10^12^ $cm^{-2}$ based on Dirac point shifting [26] and 2.5 × 10^12^ $cm^{-2}$ based on threshold voltage shifting in WSe_2_ [27]. Although the surface potential cannot be directly measured using EFM, we can estimate the change in charge density ($\Delta n_{s}$) of graphene on writing and erasing using the measured phase shift and Equation (1) [37]

(1) $$R=\frac{\Delta \Phi_{Q}}{\Delta \Phi_{C}}=-\frac{1}{3}\frac{(\frac{n_{S}}{\varepsilon_{R}})eZ}{\varepsilon_{0}\left(V_{EFM}-V_{S}\right)}$$

where *R* is the ratio between the phase shift caused by the electrostatic force ($\Delta \Phi_{Q}$) and the capacitive force ($\Delta \Phi_{C}$), $n_{S}$ is the surface charge density or sheet carrier density that results from that shift, $\varepsilon_{R}$ is the relative permittivity of graphene [38], $\varepsilon_{0}$ is the vacuum permittivity, *e* is the elementary charge, *Z* is the lift height of the tip, *V* 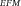 is the read bias, and *V* 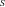 is the estimated graphene surface potential. Following Heim et al., [33] $V_{S}$ equals the tip bias that produces the minimum phase shift. For this system, $V_{S}$ equals –0.4 V at a lift height of 20 nm, as measured in Figure S1b. The phase shift on the initial read (before any writing or erasing) is $\Delta \Phi_{C}$, and the shift resulting from the repositioning of the ions by writing and erasing (i.e., the total phase shift minus $\Delta \Phi_{C}$) is $\Delta \Phi_{Q}$. This equation has been used previously, along with some geometric correction factors, to quantify charge injection into pentacene monolayer islands [33] and quantum dots [35]. Here, charge is injected and withdrawn from graphene that is equipotential with the tip, and p‐Si is the other electrode; thus, the plane capacitor form of the equation is used. The change in charge density as a function of write and erase bias is shown in Figure 4 for three heterostacks without and four with monolayer electrolyte. The data are taken sequentially (i.e., writing from –0.5 to –5 or –6 V followed by erasing from 0.5 to 5 V) The change in charge density is the change resulting from the phase shift on writing or erasing minus the phase shift before writing. The monolayer electrolyte increases the charge density by about 5 × 10^12^ $cm^{-2}$ at –4 V write bias. The result agrees well with the maximum doping density of 6.25 × 10^12^ $cm^{-2}$ for a CoCrPc to ${\mathrm{Li}}^{+}$ ratio of 1:1, based on the geometric packing density of CoCrPc as measured by scanning tunneling microscopy [24]. This result also agrees with previous measurements of the monolayer‐electrolyte‐gated graphene FETs, wherein the electrolyte added about 3 × 10^12^ $cm^{-2}$ [26].

On erasing, the change in charge density decreases with increasing erase voltage; presumably the cations are repelled by the positive erase bias, resulting in less n‐type doping. However, the total change in $n_{s}$ is less per unit applied voltage for erasing than writing. Focusing on stack 1, initially, there is a significant change in charge density on erasing, where only a small erase voltage of 0.5 V erases 5 × 10^12^ charges $cm^{-2}$. However, the subsequent change in sheet density with voltage has a weaker dependence on erasing than writing, and only partial erasing is achieved at 5 V. One possible explanation is an asymmetric energy barrier to writing and erasing. That is, after the initial ion dissipation, more energy (voltage) is required to erase than write. Per the mechanism of the monolayer electrolyte, this asymmetry corresponds to more energy required to push ions away from the graphene and toward the h‐BN (see Figure 1b(ii, iii)). Such asymmetry is plausible when considering that the ions, when positioned near the graphene, form an EDL with significantly smaller thickness (<nm) compared to the double layer thickness near the h‐BN (≈ 10 nm). As a result, the Coulomb interactions will be stronger on the graphene side, creating a larger energetic barrier to erasing. Note that we did not try larger erase biases because, as discussed above, write voltages larger than –5 V irreversibly changed the stacks, making subsequent erase measurements of equal and opposite polarity inconclusive.

We previously observed monolayer electrolyte device performance consistent with such an asymmetric energy barrier. Specifically, WSe_2_ FETs doped with the monolayer electrolyte preferred to be in the “ON state” which corresponds to the ions near the channel material, rather than the “OFF” state where they are closer to h‐BN [27]. A voltage threshold had to be reached to move ions away from the induced charge in the WSe_2_ and toward the h‐BN, providing support for the asymmetric energy barrier that is likely being detected here using EFM.

Keep in mind that all data in Figure 4 are taken at a read bias of +1 or 2 V, so the persistence of the 10^12^ $cm^{-2}$ charge during the read measurement underscores the nonvolatility of the monolayer electrolyte. This contrasts the graphene‐only heterostacks for which the change in charge density with write and erase averages 1 × 10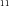 $cm^{-2}$ – about 50 times lower than the heterostacks with the monolayer electrolyte, and in good agreement with the intrinsic charge density of graphene at 300 K (3 × 10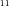 $cm^{-2}$) [39].

Note that when the heterostacks without the monolayer electrolyte are written with large biases between –3 and –6 V, the tip becomes less attracted to the surface than the before write case. These data are provided in Figure S8 and can possibly be attributed to defect generation, charge trapping and/or redistribution under write bias >3 V. In contrast, the heterostructures that contain the monolayer electrolyte can be repeatedly programmed and erased at higher voltages of ±5 V. To directly compare the heterostacks with and without the electrolyte, the total thickness of the insulating layers (i.e., h‐BN thickness, native Si oxide, and monolayer electrolyte) is accounted for to approximate a maximum electric field strength on writing and erasing. For the control stacks, the maximum field strength prior to observing irreversible changes is 0.2–0.3 V/nm, and for the monolayer electrolyte stacks, it is 0.3–0.38 V/nm. It is reasonable to expect the monolayer electrolyte to act as an electrical insulator and expand the voltage window because the bandgap is 1.34 ± 0.07 eV, as measured by scanning tunneling spectroscopy [24]. The monolayer electrolyte may extend the maximum electric field that can be applied by intoducing localized charges that screen the field. That is, a disproportionate fraction of the field drops across the monolayer electrolyte compared to the h‐BN because of the EDL.

The data above show that the written state is retained by the monolayer electrolyte for at least the duration of a 1.4 second linescan. Quantifying state retention over longer times with EFM is not straightforward because a bias must be applied during the forward scan, and we know that applying zero volts will erase the device. Thus, a small, negative forward voltage, $V_{F}$ of –0.3 V is applied that preserves more than 70% of the written state (See Figure S9a for more details). This approach allows us to write one line at –4 V, read within 1.4 s, and measure at a later time using $V_{F}$ = –0.3 followed by a read.

**Figure 5:**
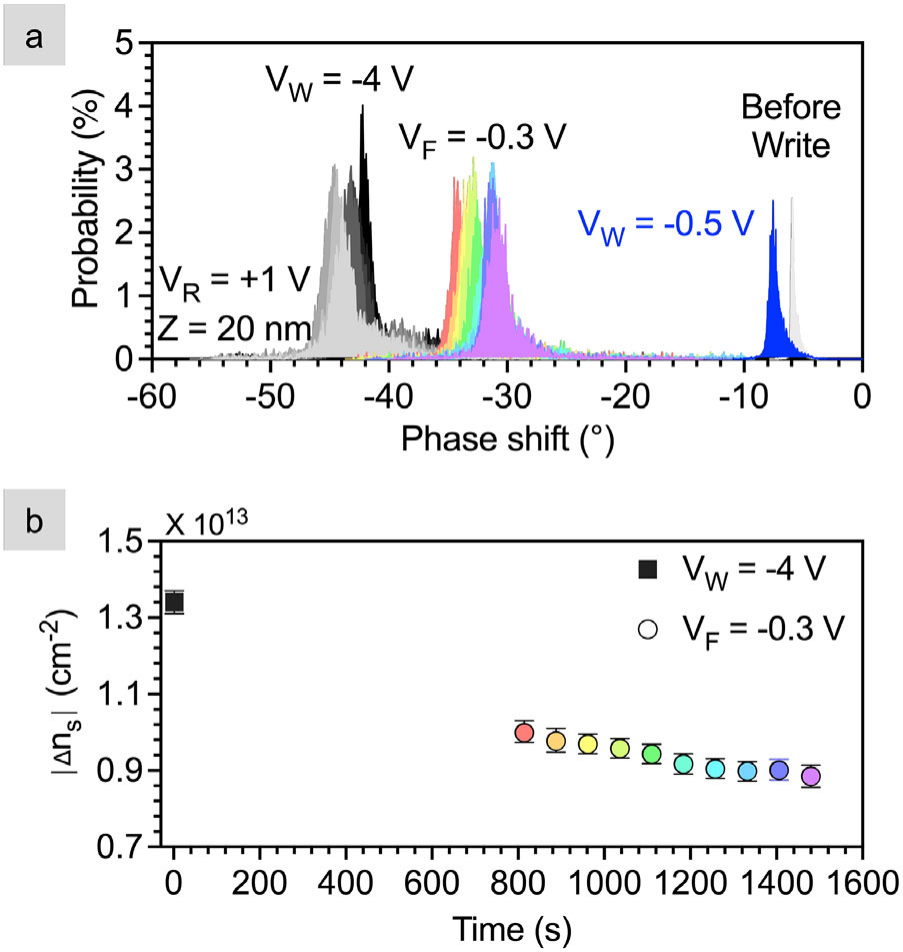
Retention measurements on stack 4 location 2. (a) Phase shift distributions read at $V_{R}$ = +1 V for each of ten sections of a 256 line scan after scanning with $V_{W}$ = –4 V and $V_{F}$ = –0.3 V. The before write distribution and low‐voltage $V_{W}$ = –0.5 V are also included for reference. (b) Change in sheet density with time. The data point for $V_{W}$ = –4 V is an average of all 10 sections; for $V_{F}$ = –0.3 V there is one data point per section. The symbol is the peak of a Gaussian fit, and the error bars represent the full width at half maximum.

To measure retention, we first write a 2 × 2 μm area with $V_{W}$ = –4 V and read the phase shift in 1.4 s. This 256 line scan takes 720 s to complete, and is followed by a second scan of the same area using $V_{F}$ = –0.3 V and $V_{R}$ = +1 V. Both scans are sectioned into ten, 2 × 0.2 μm areas, and the phase shift histograms for each section are plotted in Figure 5a. For the data read after the –4 V write, the peak location shifts $∼2^{\circ }$ and the shift is non‐monotonic; such a small phase shift is similar to the control heterostacks (Figure 3).

The second scan ($V_{F}$ = –0.3 V) began at 750 s and finished 1500 s after the write was completed. Figure 5a shows that the peak shifts 9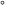 to the right between 1.4 and 750 s, and another 5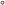 from 750 to 1500 s, indicating a loss in charge density. In contrast to the write at $V_{W}$ = –4 V, the shift to the right with $V_{F}$ = –0.3 V is monotonic; the change in charge density with time is plotted in Figure 5b. It is unclear whether the initial decrease of ∼4 × 10^12^ $cm^{-2}$ that occurs between 1.4 and 750 s represents a natural loss of charge, or if the loss is driven by the decrease in the forward sweep bias from –4 to –0.3 V. Regardless, a total increase of 9 × 10^12^ $cm^{-2}$ remains from the before write state for the duration of the measurement ∼25 min. Such state retention was also observed in the monolayer‐electrolyte‐gated WSe_2_ FETs with 2.5 × 10^12^ $cm^{-2}$ sheet density retained for at least six hours, which was the maximum time measured [27].

## 3 Conclusions

In conclusion, non‐volatile graphene doping ∼5 × 10^12^ $cm^{-2}$ is demonstrated using a molecularly thick electrolyte located between two, 2D crystals in a vertical heterostack. The non‐volatility arises from the bistability of the monolayer electrolyte. Such bistable doping originating from within the 2D heterostack brings the benefits of ion‐gating inside and not restricted to the surface of one, 2D crystal. A conductive AFM tip serves as a mobile electrode that reads, writes, erases and also senses the surface charge with a resolution of 12 × 12 nm. Using such a nanoscale contact to control the electrolyte shows promise for scaling heterostructure devices to nanometer dimensions with lithography. Such scaling is particularly relevant for the monolayer electrolyte because the state is encoded in the molecules themselves.

## 4 Experimental Section

### 4.1 Monolayer Electrolyte Deposition and Heterostack Fabrication

h‐BN was mechanically exfoliated from its bulk crystal (HQ Graphene) directly on p‐Si (University Wafer, P/Boron, 500 ± 15 μm, resistivity 1−10 ohm·cm) by the tape method (Ultron Systems, Silicone Free Blue Adhesive Plastic Film). Si substrates were cleaned with acetone and isopropanol (IPA) before exfoliation. Exfoliated h‐BN flake thicknesses between 7 to 15 nm were measured by AFM Peakforce Tapping (Bruker Dimension Icon) with Si_3_N_4_ ScanAsyst‐air tips (0.4 N/m). The flakes were annealed at 240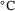 for 30 min.

CoCrPc was deposited onto h‐BN in an Ar‐filled glovebox with H_2_O and O_2_ concentrations <1 ppm following a procedure similar to previous publications [24, 26, 27, 28]. CoCrPc was dissolved in a 9:1 v/v anhydrous benzene/ethanol mixture (Sigma–Aldrich, 99.8%/99.5%) at a concentration of 13 mg/L. 46 μL was drop‐cast onto a ∼1 × 1 cm substrate with a micro‐pipette and annealed at 240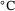 for 30 mins to evaporate the solvent. AFM topology measurements were made before and after deposition to confirm a monolayer of CoCrPc. Next, LiClO_4_ was dissolved in anhydrous ethanol at a concentration of 1 mg/L. 48 μL was drop‐cast onto the substrate and annealed at 180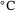 for 30 min. The molar ratio of CoCrPc to ${\mathrm{Li}}^{+}$ was maintained at 1:1, consistent with our previous publications [27].

Graphene was mechanically exfoliated from highly ordered pyrolytic graphite (HOPG) onto p‐type Si with 90 nm thermally grown SiO_2_ (Graphene Supermarket, resistivity 0.001−0.005 ohm·cm). Graphene flakes of thickness ∼2 − 3 nm were transferred in a glovebox by a polycarbonate/polydimethylsiloxane (PC/PDMS) stamp. Using a micromanipulator and optical microscope, the stamp was aligned over the electrolyte, lowered into contact with the substrate and heated to 185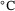 to release the PC from the PDMS. The residual PC was removed by dissolving it in chloroform.

### 4.2 Direct Write, Read, and Erase with EFM

Heterostacks were imaged and measured in EFM mode using a Bruker Dimension Icon AFM housed in an Ar‐filled glovebox. Measurements were made using a conductive AFM tip (SCM‐PIT‐V2, Bruker Nano, 3 N/m) using a two‐pass technique. In the first pass, known as the feedback pass, the topology of the heterostack was recorded through tapping‐mode AFM. In the second pass, called the interleave pass, the tip was fixed at a distance between 20 and 25 nm from the surface to sense only the electrostatic interactions between the tip and the surface. All topology and phase image scans were analyzed using Bruker's Nanoscope 1.8 software.

### 4.3 Retention Measurements

The voltage for the forward scan ($V_{F}$), which is required to read the written state, was determined using the following procedure, where $V_{R}$ = +1 V for all retention measurements. A 2 × 2 μm region was written with a –0.5 V write bias, resulting in a ∼2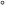 phase shift, followed by a –4 V bias, resulting in a ∼35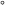 phase shift. These small and large phase shifts will be comparable to those measured for Stack 3, Location 2, shown in Figure 2c, demonstrating consistent measurements across heterostacks. Once the area was written at –4 V, $V_{F}$ was identified by applying a small voltage (between –0.1 and –0.3 V) to the same area, followed by a read at +1 V. If the small $V_{F}$ fully disturbed the written state, then the phase shift would be positioned closer to that of the –0.5 V write data. However, if it did not disturb the state, the phase shift would be more similar or equal to the phase shift after the –4 V write.

## Conflicts of Interest

The authors declare no conflicts of interest.

## Date Availability Statement

The data that support the findings of this study are available from the corresponding author upon reasonable request.

## Supporting information


**Supporting File**: smll72746‐sup‐0001‐SuppMat.pdf.
