# Supplementary material for: Direct Write, Read, and Erase of a Vertical Heterostructure of Graphene–Monolayer Electrolyte–h‐BN Using Electric Force Microscopy
Source: Small. 2026 Feb 20;22(20):e11129. doi: 10.1002/smll.202511129 (PMC13054451; doi:10.1002/smll.202511129)
Supplement: Supplementary file 1 — Supporting File: smll72746‐sup‐0001‐SuppMat.pdf. [file SMLL-22-e11129-s001.pdf]

Supporting Information – Direct Write, Read, and Erase of a Vertical Heterostructure of Graphene–Monolayer Electrolyte–h-BN Using Electric Force Microscopy

Huiran Wang,[†] Shubham Sukumar Awate,[†] Ke Xu,[‡,§,†] and Susan K. Fullerton-Shirey[*,†,∥,⊥]

†*Department of Chemical and Petroleum Engineering, University of Pittsburgh, Pittsburgh, Pennsylvania 15260, United States*

‡*School of Physics and Astronomy, Rochester Institute of Technology, Rochester, New York 14623, United States*

¶*School of Chemistry and Material Science, Rochester Institute of Technology, Rochester, New York 14623, United States*

§*Microsystems Engineering, Rochester Institute of Technology, Rochester, New York 14623, United States*

∥*Department of Electrical and Computer Engineering, University of Pittsburgh, Pittsburgh, Pennsylvania 15260, United States*

⊥*Current address: 3700 O'Hara Street, Pittsburgh, Pennsylvania 15213, United States*

E-mail: fullerton@pitt.edu

1 Determining Lift Heights and EFM Voltages

The tip lift height and read bias were determined as follows. An h-BN flake was exfoliated onto p-type Si followed by exfoliated graphene, which was fully supported by the h-BN. A 3×3 μm area was selected that included the step interface between h-BN and graphene. The tip bias is was set to 0 V for both passes and the lift height (Z) was increased from 0 to 75 nm until the phase difference between hBN region and the graphene/h-BN was no longer discernible, and the phase shift became independent of lift height. As shown in Figure S1(a), the phase shift becomes largely independent of lift height at values greater than 10 nm. We choose a 20 nm lift height to minimize Van der Waals forces and ensure that the tip interaction with the surface is dominated by electrostatic forces. Such a lift height is within a common range for sensing electrostatic forces (15 – 50 nm).[1,2]

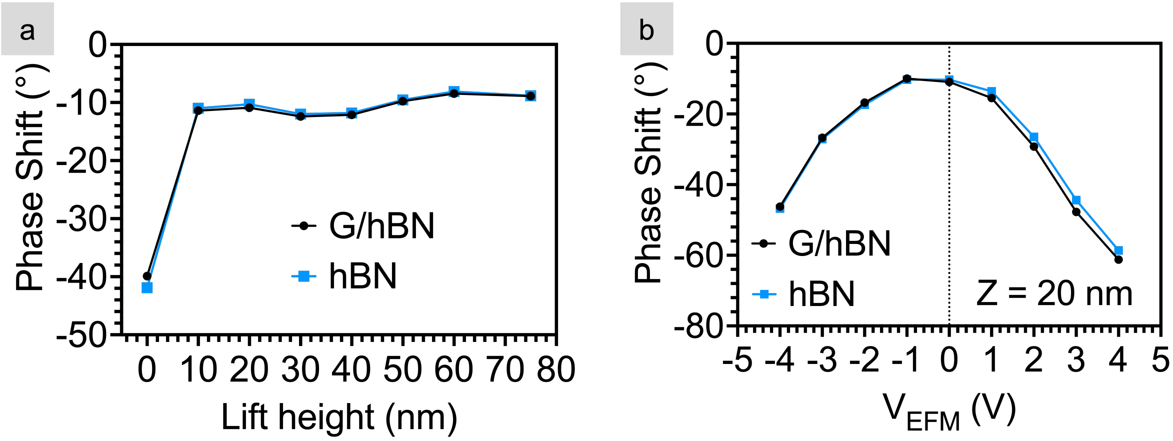

Figure S1: Lift height and bias calibration on graphene and h-BN heterostack. Phase shift as a function of **(a)** tip lift height (z) and **(b)** tip voltage (V_{EFM}) when the tip is located 20 nm from the h-BN surface or the graphene/h-BN heterostack.

To determine the read bias, we identify a voltage at a given lift height (in this case, 20 nm), for which the phase shift depends weakly on V_{EFM}. The read bias should sense the charge distribution but not disturb it – especially in the case of the monolayer electrolyte. Thus, the feedback pass is set to 0 V, the interleave pass, the lift height is fixed at 20 nm and the V_{EFM} voltage is varied from –5 to 5 V, shown in Figure S1(b). Voltages greater than

± 1 V show a significant change in the phase shift with increasing voltage. Specifically, for $+1$ V read, we can discern a modest (approximately 2 degree) difference in the phase shift between the surface of graphene/hBN and h-BN. This could be interpreted as a resolution of the read measurement under positive bias, because the phase shift is able to detect small differences in the surface. This is not the case for negative read biases. Thus, we choose $+1$ V as the read voltage because it is the smallest non-zero voltage for which some differences in the charge distribution at the two surfaces can be detected. In some measurements of additional stacks, we also try a read voltage of 2 V (see Figures S6 and S7 below).

2 Phase Shift Response to Write Voltage of −4 V

Figure S2 shows that the large phase shift shown in the main document (Figure 2c) can be achieved by directly applying $V_W = -4$ V, and does not require the application of progressively larger voltages.

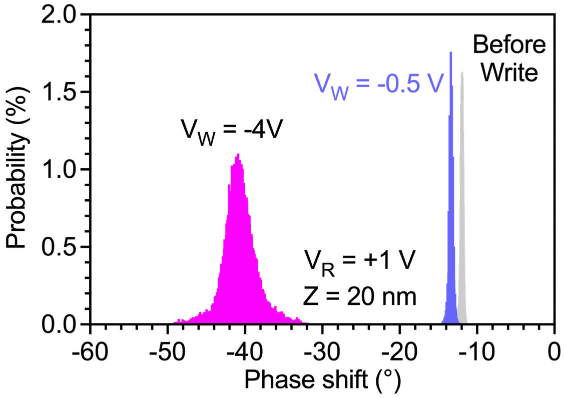

Figure S2: Phase shift histogram showing the response after direct application of $V_W = -4$ V with $V_R = +1$ V and $Z = 20$ nm.

3 AFM and EFM Scans

Figure S3(a) and (b) are AFM topographical scans of the same area (Stack 1, Location 1) before write and after $V_W = -4$ V. Note that this is the same stack and scan location as illustrated in Figure 2(b). The similarity between the scans indicates that topology is not strongly impacted by a large and negative write voltage.

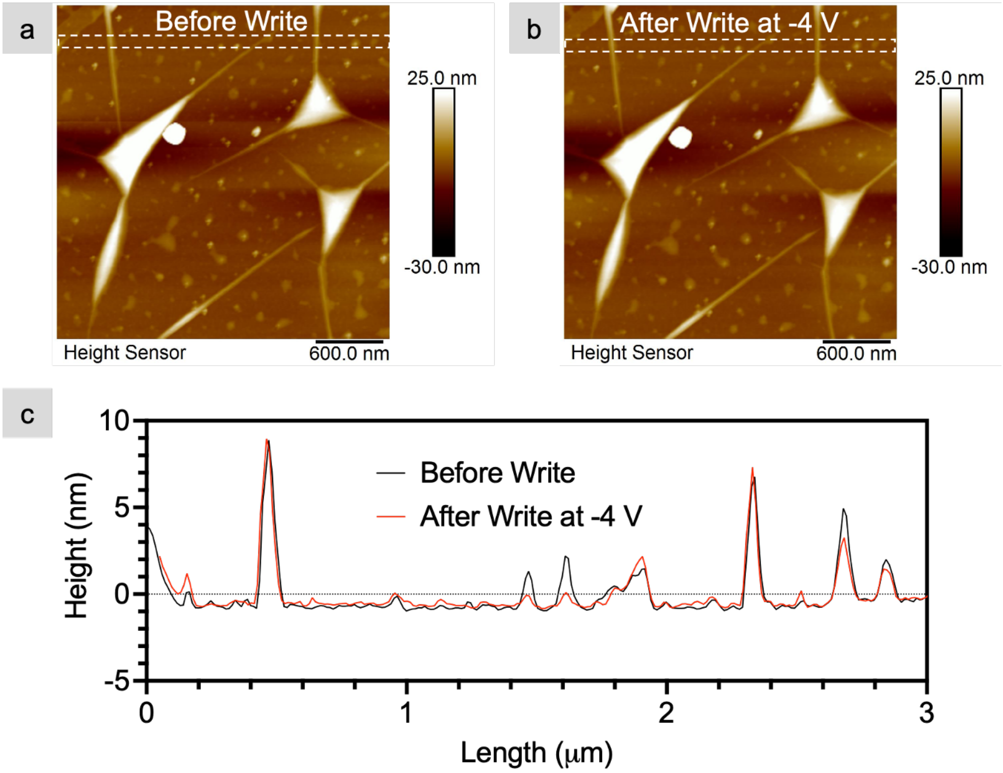

Figure S3: AFM topographical scans of the same area **(a)** before write, and **(b)** after write at –4 V. The white boxes, with dimensions of 3 μm by 100 nm corresponding to the average of 9 linescans, in **(c)** are taken at the top of the scans in the area indicated by the white boxes. This area is selected from Stack 1, Location 1.

The histograms in Figure 2(c,f) and 3(c,f) in the main text summarize the phase shift data extracted from Figures S4 and S5.

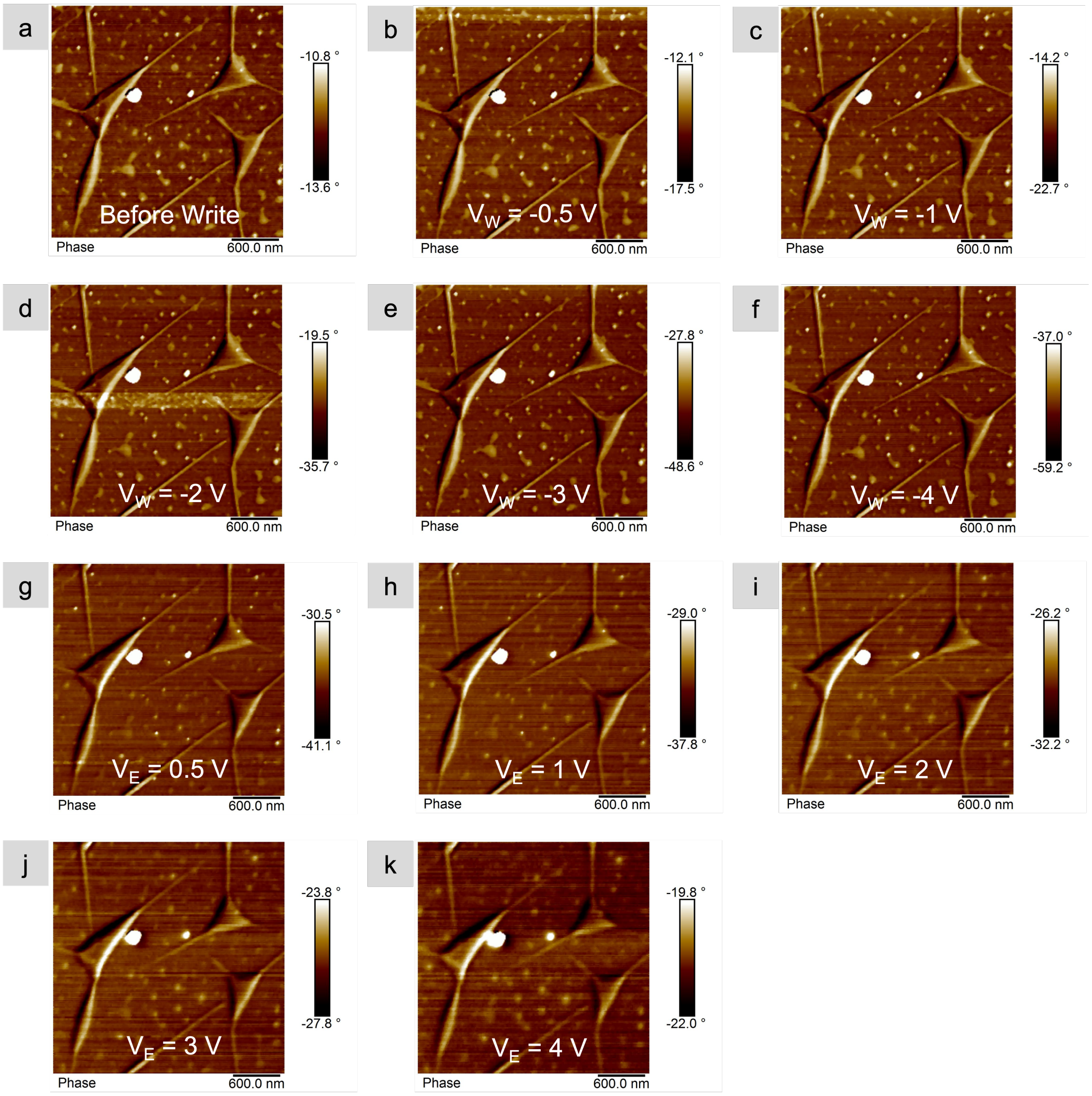

Figure S4: EFM Phase images of a 3 $\times$ 3 μm region on heterostack 1 (Location 1) containing the monolayer electrolyte (i.e., G/ME/h-BN). Z = 20 nm and $V_R = 1$ V. **(a)** Read measurement prior to writing or erasing. The same region **(b - f)** written with the feedback pass set to $V_W = -0.5, -1, -2, -3,$ and -4 V, and **(f - k)** erased with $V_E = 0.5$, 1, 2, 3 and 4 V.

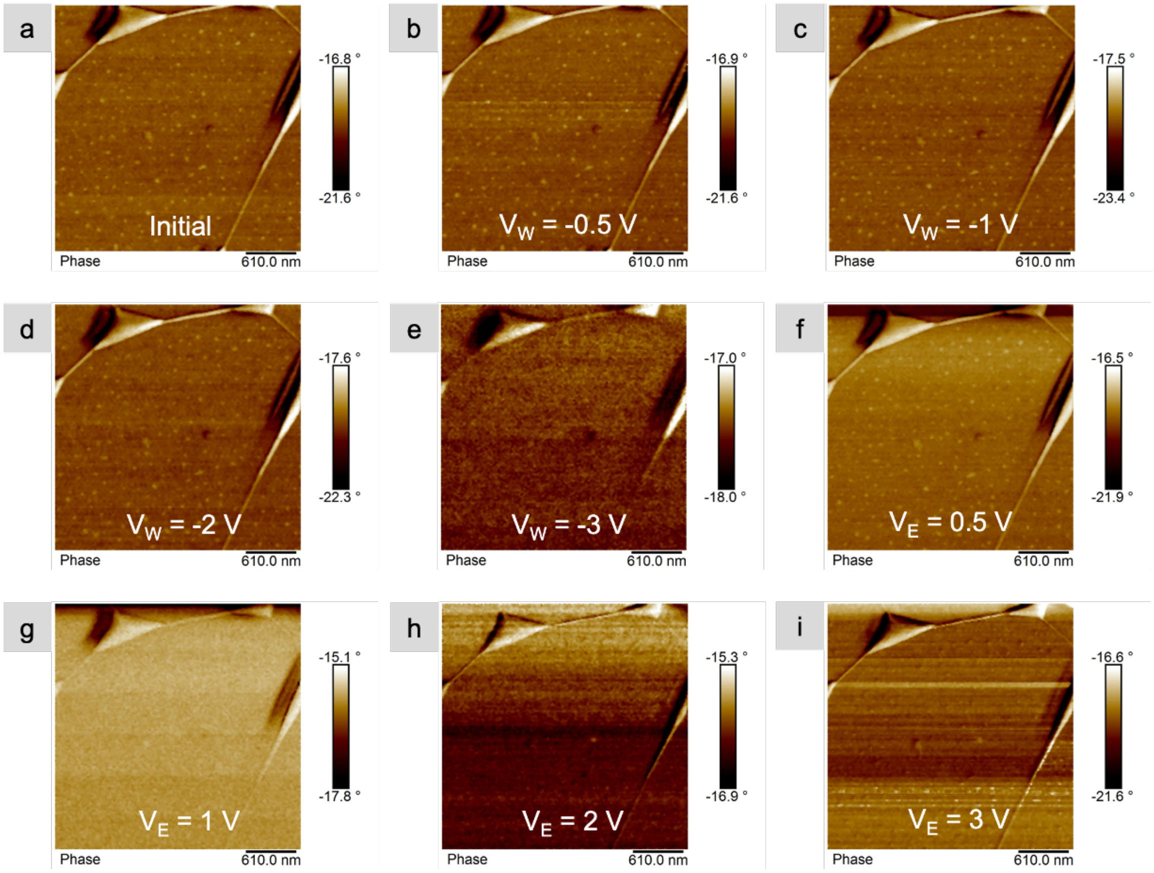

Figure S5: EFM Phase images of a 3×3 μm region on heterostack 1 without the monolayer electrolyte (i.e., G/h-BN). Z = 20 nm and $V_R = 1$ V. **(a)** Read measurement prior to writing or erasing. The same region **(b - e)** written with the feedback pass set to $V_W = -0.5$, -1, -2, and -3 V, and **(f - i)** erased with $V_E = 0.5$, 1, 2, and 3 V.

4 EFM Measurements of Additional Heterostacks

In addition to the heterostacks in the main document, we assembled and measured three additional stacks containing the monolayer electrolyte and two additional stacks without. We also measured multiple locations on those stacks. The phase shift histograms of those additional stacks are shown in Figures S6 for the monolayer heotrostack and Figure S7 for the control stack. While the absolute values of the phase shifts may vary between stacks and locations, the trend remains the same as described in the main text.

The stack-to-stack and location-to-location variability is quantified in the main document, Figure 4. Specifically, S1, S2, S3 and S4 in Figure 4 correspond to the data taken for Stacks 1 through 4 in Figure S6. For the control samples (i.e., without monolayer electrolyte), C1, C2 and C3 in Figure 4 correspond to the data taken for Stacks 1 through 3 in Figure S7.

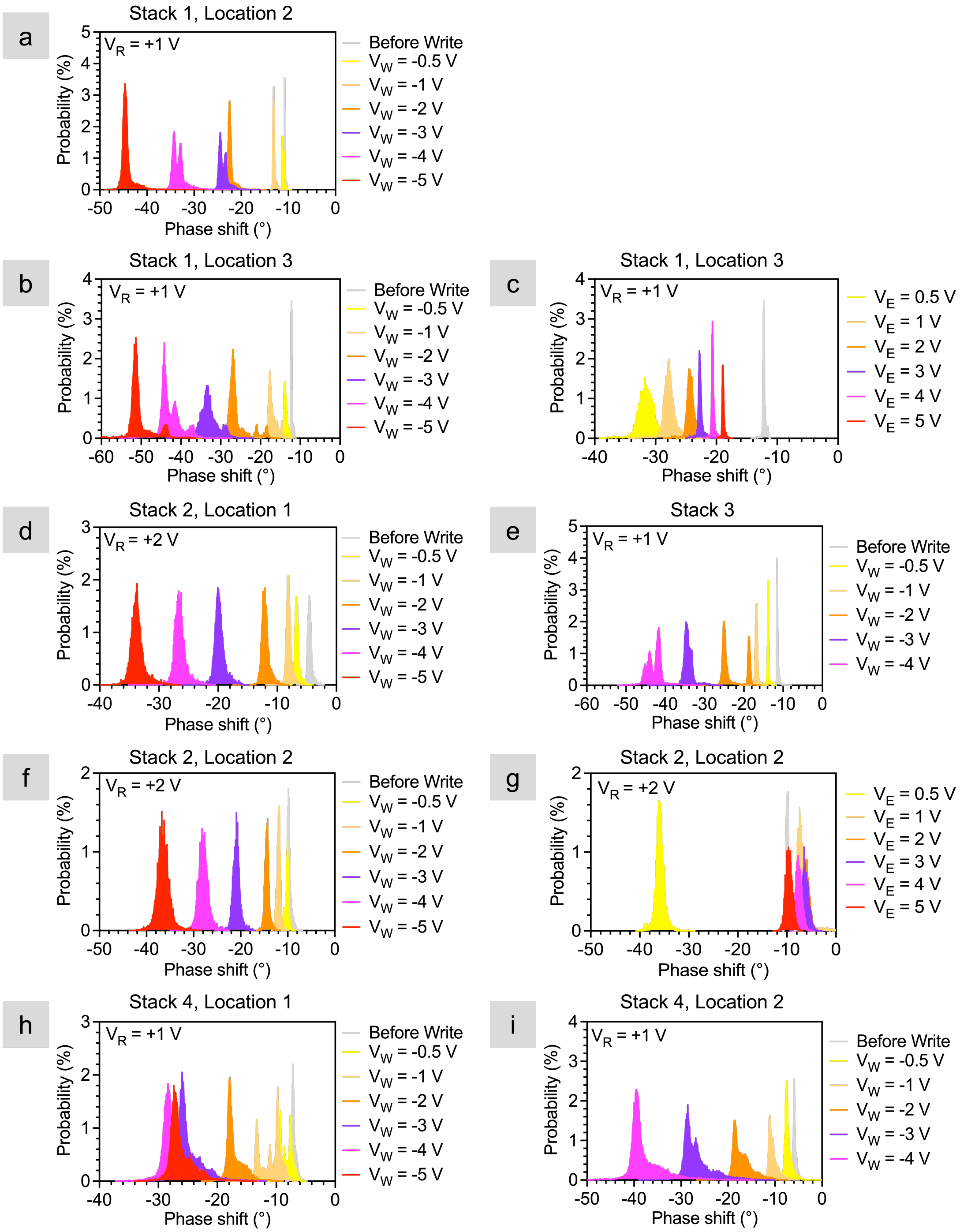

Figure S6: Phase shift histograms measured on multiple heterostacks at multiple locations for the heterostacks containing the monolayer electrolyte (i.e., graphene/monolayer electrolyte/h-BN). Writing **(a)** stack 1, location 2 and **(b)** stack 1, location 3. **(c)** Erasing stack 1, location 3. Writing **(d)** stack 2, location 1 and **(e)** stack 3. Writing **(f)** and erasing **(g)** stack 2, location 2. Writing **(h)** stack 4, location 1 and **(i)** location 2. V_R for d, f, and g equals +2 V and +1V for all other measurements; Z = 20 nm.

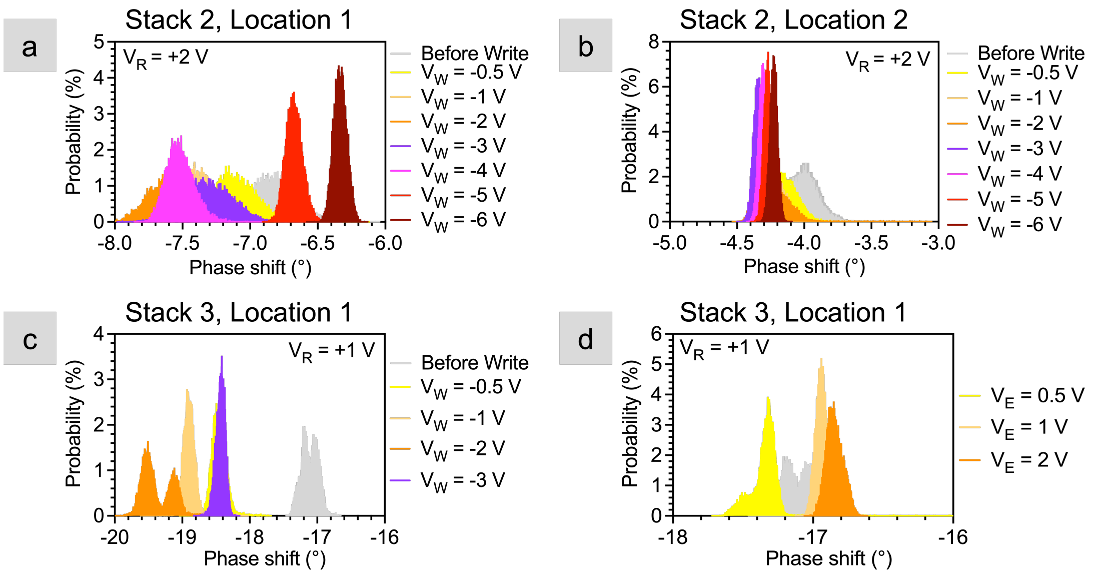

Figure S7: Phase shift histograms measured on multiple heterostacks at multiple locations for the control heterostacks (i.e., graphene/h-BN). Writing **(a)** stack 2, location 1 and **(b)** stack 2, location 2. Writing **(c)** and erasing **(d)** stack 3, location 1. V_R for a and b equals +2 V while V_R equals +1 V for c and d; Z = 20 nm.

Notice that in the graphene/h-BN control stacks, when a large and negative voltage (i.e., $V_W > -3$ V) is applied, the phase shift moves in the more positive direction, and in some cases, it shifts to the right of the "Before Write" value. This physically means that the tip, with a +1 V read bias, is now less attracted to the surface than prior to any charge injection. It is not possible to know for sure from these data, but one possible explanation is that large voltages damage the graphene and potentially drive chemical changes at the surface corresponding to defect generation. In fact, when these control samples that have experienced > -3 V write voltages are remeasured, the phase shifts are universally in the positive direction, as shown in S8 for Stacks 2 and 3. These results suggest an upper voltage limit of -4 V.

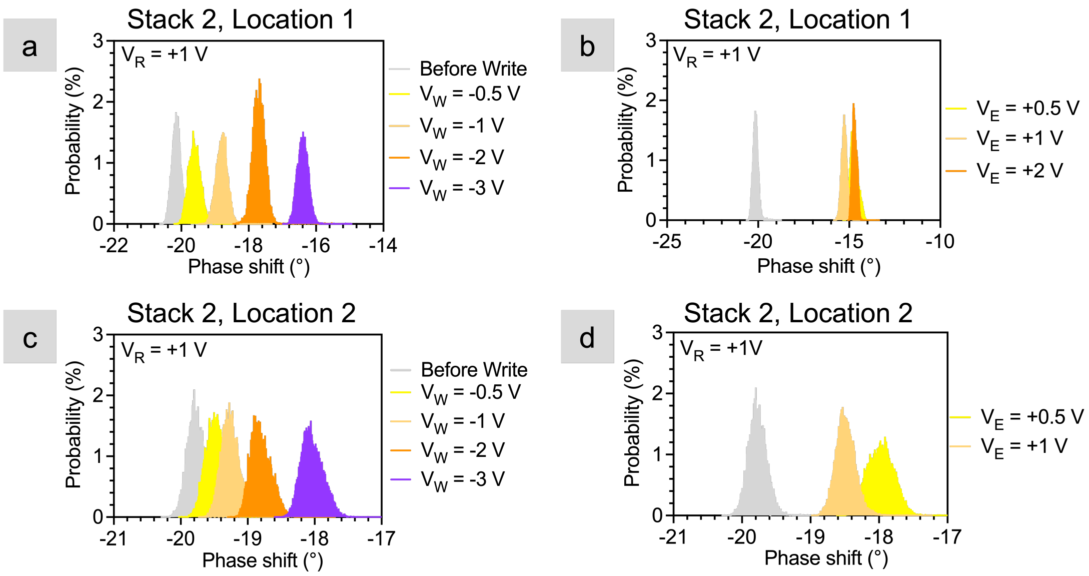

Figure S8: Write and erase phase shift histograms for remeasured control stacks 2 and 3 (G/hBN) after they experienced a high-voltage write step (–6 V). Stack 2 location 1 **(a)** write and **(b)** erase. Stack 2 location 2 **(c)** write and **(d)** erase.

5 Retention Measurements

The voltage for the forward scan (V_F), which is required to read the written state, was determined using the following procedure. (Note that the read voltage (V_R) is equal to +1 V in all measurements.) A 2 × 2 μm region was written with a –0.5 V write bias, resulting in a $\sim$ 2° phase shift, followed by a –4 V bias resulting in a $\sim$ 35° phase shift, and shown in Figure S9(a). These small and large phase shifts are comparable to those measured for Stack 3, Location 2, shown in Figure 2(c) of the main manuscript, demonstrating consistent measurements across heterostacks. Once the area was written at –4 V, V_F was identified by applying a small voltage (between –0.1 and –0.3 V) to the same area, followed by a read at +1 V. If the small V_F fully disturbed the written state, then the phase shift would be positioned closer to that of the –0.5 V write data. However, if it did not disturb the state, the phase shift would be more similar or equal to the phase shift after the –4 V write.

As shown in Figure S9(a), using V_F values between –0.1 and –0.3 V does result in the loss of about 10° of the original phase shift; however, it remains about 22° shifted from the original location. That is, if V_F fully disturbed the written state, the resulting phase shift would be positioned between the "Before Write" and V_W = –0.5 V data at –7°; however, it is positioned around –30°.

It is unclear whether or not this loss is the natural consequence of state volatility, or if the loss is driven by V_F itself. Regardless, because the device retains more of the state than it potentially disturbs, we regard V_F = –0.3 V as sufficient for exploring state retention in the heterostacks.

To get some sense for retention, we started by writing regions of progressively larger area with –4 V, followed by scans with V_F = –0.3 V. This approach allows us to change the retention time by changing the scan area. Specifically, the scan time required for each area (quantified as number of lines) are provided in Table S1. The aspect ratio of the scans (i.e., width/length), is also provided. Note that the same number of data points are acquired per

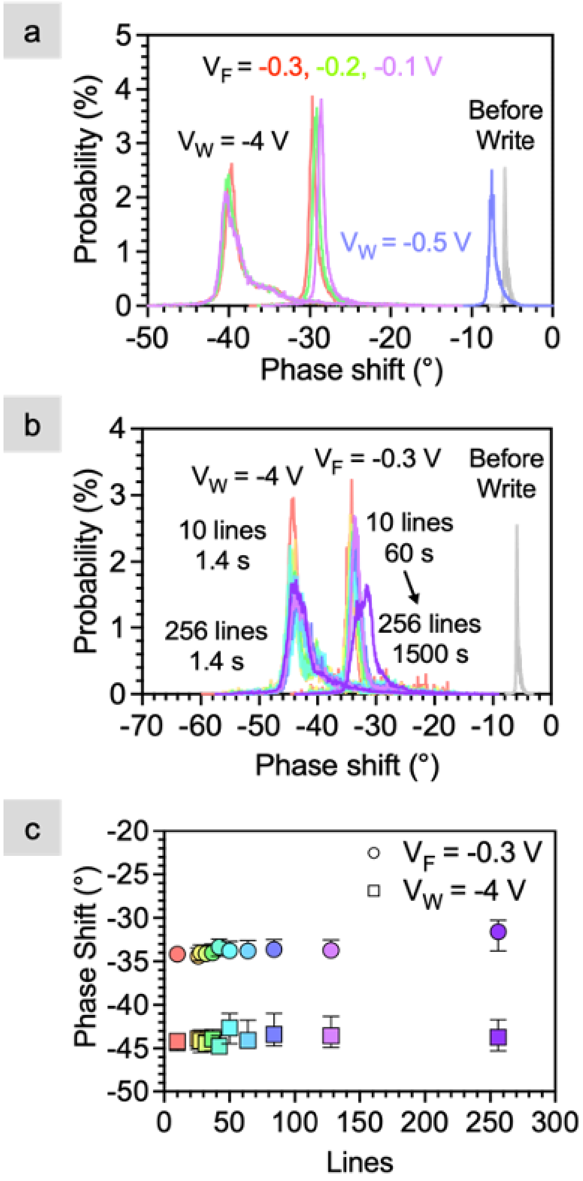

Figure S9: (a) Histograms used to confirm that a V_F = –0.3 V is sufficient to read the state without entirely erasing it, because the peaks labeled V_F do not return to the "Before Write" peak. (b) Histograms with varying scan size from 10 to 256 lines. In the case of V_W = –4 V, the time between write and read remains 1.4 s regardless of scan size. However, for V_F = –0.3 V, the time between writing and scanning with V_F varies between 60 and 1500 seconds. (c) Phase shift plotted as a function of the scan lines. The location of the peaks correspond to the location of the data point and the error bars indicate the full width at half max of each distribution. These measurements are performed on Stack 4, location 2 (with monolayer electrolyte).

line, and only the total number of lines varies. These scan times listed are for one scan; however, two scans (the first at V_W = –4 V and the second at V_F = –0.3 V) are measured.

Thus, the total scan time varies from 10 lines which requires 60 seconds to 256 lines which requires 1500 seconds.

Table S1: Aspect ratio of the EFM scan (width/length), number of lines scanned, and total scan time in seconds.

Aspect Ratio	Number of Lines	Scan time (s)
25	10	29
10	26	75
9	28	81
8	32	93
7	37	107
6	43	124
5	50	147
4	64	185
3	85	146
2	128	370
1	256	740

The phase shift data resulting from this procedure are shown Figure S9(b). First, focusing on the collection of peaks around –44° labeled "V_W = –4 V". These measurements are each taken within 1.4 s of writing at –4 V, and therefore the broadening peak distribution reflects the inhomogeneity in the sample as the scan area increases and more data are collected. For the collection of peaks near –34° labeled "V_F = –0.3 V", the distributions become broader for presumably the same reason, but the peak locations also shift to the right moving from scans that take 60 to 1500 s. This shift represents a loss of state retention as the graphene becomes less n-type.

The phase shift with number of lines for both V_W = –4 V (i.e., 3 seconds of retention) and V_F = –0.3 V (i.e., 60 to 1500 seconds) of retention are plotted in Figure S9(c). Here, we can see that the peak of the phase shift remains largely unchanged at 3 seconds, but drifts several degrees after 1500 seconds.

References

(1) Burnett, T.; Yakimova, R.; Kazakova, O. Mapping of local electrical properties in epitaxial graphene using electrostatic force microscopy. *Nano Letters* **2011**, *11*, 2324–2328.

(2) Li, L. H.; Chen, Y. Electric contributions to magnetic force microscopy response from graphene and MoS2 nanosheets. *Journal of Applied Physics* **2014**, *116*.
